# Validation of the professional identity questionnaire among medical students

**DOI:** 10.1186/s12909-021-02704-w

**Published:** 2021-06-28

**Authors:** Daan Toben, Marianne Mak-van der Vossen, Anouk Wouters, Rashmi A. Kusurkar

**Affiliations:** 1 Faculty of Health and Life Sciences, Vrije Universiteit Amsterdam, Polanenstraat 54F, 1013 VZ Amsterdam, The Netherlands; 2 Amsterdam UMC, Faculty of Medicine Vrije Universiteit Amsterdam, Research in Education, Amsterdam, the Netherlands; 3 LEARN! Research Institute for Learning and Education, Faculty of Psychology and Education, VU University Amsterdam, Amsterdam, the Netherlands

**Keywords:** Construct validity, Designing and evaluating medical curriculum, Medical students, Professional identity, Structural equation Modelling, Confirmatory factor analysis, Exploratory factor analysis

## Abstract

**Background:**

Professionalism represents a cornerstone of the medical profession, prompting medical educators to actively develop instruments to measure professional identity formation among medical students. A quantitative approach to this problem has been lacking. Hence in this study, we investigate the validity and reliability of using Brown et al.’s [1986] Professional Identity Questionnaire (PIQ) to measure professional identity among medical students.

**Methods:**

We used the American Psychological Association’s account of validity and reliability to examine the PIQ in terms of its internal structure, its relation to a validated motivation scale, its content, and its internal consistency. To this end, we performed two factor analyses, a Pearson’s correlation test, an expert evaluation and measured Cronbach’s alpha, respectively..

**Results:**

Factor analysis revealed two latent factors underlying the items of the PIQ. We found a negative to positive spectrum of Pearson’s correlations corresponding to increasingly internal qualities of motivation. Experts unanimously rated four out of ten of the PIQ’s items as relevant, reliability analysis yielded a Cronbach’s alpha value of 0.82.

**Conclusion:**

Despite poor ratings by experts in the field, these results illustrate the PIQ as a valid and reliable quantitative measure of medical students’ professional identity; its two factors reflecting the measure of attached and detached attitudes towards the medical profession. Educators may use the instrument as a tool for monitoring PIF among their students, as well as for designing and evaluating their medical curriculum. Future research might build on the current findings by investigating other dimensions of the PIQ’s validity, including response process validity, predictive validity and consequential validity.

**Supplementary Information:**

The online version contains supplementary material available at 10.1186/s12909-021-02704-w.

## Introduction

Professionalism is one of the core competencies within the medical profession [1]. In their report on the education of the next generation of health professionals, Frenk et al. [2] called for a renewed focus on professionalism. However, there exists confusion on its definition. Irby & Hamstra [3] ascribe this confusion to the simultaneous use of three frameworks for professionalism within the medical education professionalism discourse. One framework is virtue-based, with a focus on moral character, moral reasoning and humanism. Another is behaviour-based, focusing on competencies and behavioural milestones. A third framework focuses on the development of professional identity. This study focuses on the professional identity formation framework, emphasizing the evolution of a professional identity through developmental processes of being and becoming [3]. Medical educators are actively developing and implementing measures for professional identity formation (PIF) among medical students throughout their medical study [4]. However, to date there is no available evidence for an instrument that measures PIF in a valid and reliable way among medical students. The development of such a tool could aid the medical educational system by allowing educators to assess how effectively their curriculum, its formal, informal and hidden parts, instil professional identity in their students. Brown et al. [5] developed a tool called the Professional Identity Questionnaire (PIQ) for the quantitative measurement of professional identity. This tool appears promising; the authors reported a reasonable measure of internal consistency represented by a Cronbach’s alpha value of 0.71 and made a case for its validity by comparing the scores of participants who were positively affected towards their vocation versus those negatively so. They found scores of these two groups to differ significantly. Nevertheless, such evidence is insufficient in and of itself and the scale’s use has yet to be researched in a group of medical students. This study, therefore, aims to build on Brown et al.’s [5] case by investigating if the PIQ can be used for the valid and reliable measurement of professional identity among medical students [5].

Professional identity funnels into professionalism at the apex of Miller’s pyramid as being, rather than solely knowing, knowing how, showing how, and doing [4, 6]. Research on how to most effectively foster the formation of professional identity is scarce [7]. Mylrea, Gupta & Glass [8], albeit specifically for pharmacy majors, stress the need for experiential learning with regards to professional identity formation. They specifically endorse increased contacts between students and practising professionals as a method of developing students’ competence, relatedness and autonomy. Using the Self-Determination Theory [9] these authors describe the development of these three basic human psychological needs as a driver of professional identity formation through their internalizing the motivation for the enactment of the new role. This concurs with findings reported by Tagawa [10], whose validation inquiry of another PIF scale for Japanese medical students uncovered internalization of values as one of its five main factors. Interestingly, Kalet et al. [11] reported various individual patterns that deviated from this trend of internalization, which they ascribed to the non-linear nature of identity development in general. That is, because professional identity formation progresses, or even regresses, in fits and starts that alternate long periods of stability, the snapshot picture created through measurement is likely to reveal some idiosyncrasies.

Therefore, the complexity of professional identity as a construct warrants a variety of tools for its measurement. The availability of a valid and reliable quantitative measure could open up avenues for research and further understanding of PIF, as it would facilitate comparisons of professional identity formation across scientific paradigms and nationalities. The current study, therefore, aims to validate Brown et al.'s [5] PIQ using the account of validity developed jointly by the American Psychological Association (APA), the American Educational Research Association and the National Council on Measurement in Education (NCME) [12]. In this account, construct validity is described as the central focus of validation work [13], which lends credit to the specified interpretation and use of the scores of the instrument. This study assesses the PIQ’s construct validity through an accumulation of three types of validity evidence as well as one type of reliability evidence: validity in terms of content, internal structure, relations, and reliability in terms of internal consistency. Content validity reflects the degree to which the instrument’s items represent all facets of the construct. Internal structure validity reflects the extent to which the relationship between the instrument’s items represent the construct it is intended to measure. Concurrent validity reflects the degree to which the scores of the instrument relate to scores of an instrument that validly measures a similar construct. And internal consistency reflects the degree to which the instrument’s items are homogenous. Four hypotheses are consequently tested: a) Relating to content validity, the PIQ will receive a synonymous high scoring by experts using a content validity index; b) Relating to internal structure validity, the items on the PIQ measure one latent factor, namely professional identity; c) Relating to concurrent validity, and in accordance with the Self-Determination Theory of motivation, total score on the PIQ will correlate positively with the more self-determined/autonomous types of motivation and negatively with less self-determined/controlled types of motivation; and d) Relating to internal consistency, the PIQ’s score reflects a reliable measure of the construct of professional identity.

## Methods

### Participant selection

The current study utilized a part of the dataset of the Student Motivation and Success (SMS) study, a longitudinal study of academic motivation, learning strategies, engagement, empathy, personality and academic as well as professional achievements of students of medicine at the Faculty of Medicine VU Amsterdam [14]. The PIQ (Additional file 1: Appendix A) was filled out by students from all three Bachelor years and all three Master years of medicine in the years 2015, 2016 and 2017 at the beginning of the academic year. Participation was voluntary and written informed consent was obtained from all participants.

### Ethical approval

Ethical approval was granted for this study by NVMO-ERB (Netherlands Association for Medical Education - Ethical Review Board), folder # 1041 (amendment to dossier #388).

### Instrument

The PIQ was developed by Brown et al. [5] to measure the extent of social identification of an individual with a certain group. Specifically, it has been used to assess the degree to which nurses working in various hospitals in South England identify with their specialized vocational groups [15]. It contains 10 items to be scored on a 5-point Likert scale, with 1 being “never” and 5 being “very often”. Items F through J were identified as being negatively formulated and, as such, the scoring on these items was reversed. A total score representing a quantitative measure of professional identity was consequently computed. Both above-mentioned studies have reported an acceptable level of reliability (Cronbach’s α = 0.77) for the PIQ.

### Statistical analysis

IBM SPSS version 24.0 was used for statistical analyses along with the lavaan module within R [16].

#### Content validity

Experts on the subject of professional identity formation in undergraduate medical students were consulted to verify the questionnaire’s content validity. Selection occurred through snowball-sampling, using the SMS study as a basis of expertise. All four experts were seasoned researchers in the field of professional identity and were specifically selected as such, having multiple publications on the subject to their name. Correspondence with the experts was carried out by email. They received the instrument itself as well as an elucidation of its contents. The experts were asked to use the Inter item Content Validity Index (I-CVI) [17], administered through the internet-based survey programme Qualtrics. The experts assessed each item’s relevance on to a four-point scale (1 = not relevant to 4 = highly relevant). The four-point scale was chosen because it omits the option of a neutral answer that could have a strong diluting effect on the results in a small pool of experts. The results of the I-CVI were then processed as the number of experts giving a score of 3 and 4 divided by the total amount of experts, thereby arriving at an index number. As a cut-off point, an I-CVI index of 1.00 was used to distinguish relevant from non-relevant items in accordance with the literature [17].

#### Internal structure validity & measurement invariance

Factor analysis was used to assess the questionnaire’s internal structure validity after checking if the data were normally distributed. Exploratory Factor Analysis (EFA) was conducted on the 2015 dataset. Assumptions were checked using a variety of measures, their cut-off points were based on existing literature [18]: The Kaiser-Meyer-Olkin measure (KMO) was employed, utilizing a cut-off point of 0.50; Kaiser’s criterion for eigenvalues of 1.0 was used as a cut-off point for factors; Bartlett’s test of sphericity was used to determine the goodness of fit of the model, using a cut-off point of 0.50. The Principal Axis Factoring method was chosen as an appropriate approach as it seeks the least number of factors to describe common variance among the variables (75–85%) [19, 20]. A cut-off point of 0.30 was used to distinguish weak from strong factor loadings, in accordance with the literature [21]. When it came to cross loading a cut-off point of 0.32 was used, also in accordance with the literature [22]. Additionally, the Varimax method of rotation was used. A Confirmatory Factor Analysis (CFA) was subsequently performed on the 2016 dataset. This analysis was conducted using the Lavaan package within R [16]. Model fit for the CFA was assessed by calculating the Tucker-Lewis Index (TLI), the Comparative Fit index (CFI), the Root Mean Square Error of Approximation (RMSEA) and the Standardised Root Mean square Residual (SRMR) [23].

The CFA model thus constructed was also used to assess measurement invariance through multiple group confirmatory factor analysis [24]. That is, it was used to assess whether the PIQ is interpreted differently across groups. In this study, we differentiated on the basis of gender in order to test whether the PIQ’s measure of professional identity is equal between women and men. As a dichotomous variable, gender fits well with the relatively small sample size of this study. Furthermore, as the PIQ is intended to measure PIF in a group – medical students – comprising both males and females, it is important to verify psychometric equivalence across them. In a stepwise fashion representing ever increasing constraints that are imposed upon the model, the CFA model was tested on the basis of three hypothesized levels of equivalence between these two groups [24]. Briefly stated, configural equivalence holds that the hypothesized factor structure of the measure is equal between groups. Metric equivalence holds that how the items load unto these factors be equal between groups. Lastly, scalar equivalence holds that the intercepts of each item be equal between groups. At each step, the model fit indices described by [23] were used as indicators of non-equivalence [24].

#### Concurrent validity

To assess the questionnaire’s concurrent validity the total score of the PIQ was compared to that of a questionnaire measuring quality of motivation, the Academic Self-regulation Scale (SRQ-A) (Additional file 1: Appendices B-I & B-II) [25, 26]. The SRQ-A is based on the Self-Determination Theory of motivation [9, 27]. It is comprised of subscales measuring four regulations of extrinsic (EM) and intrinsic (IM) qualities of motivation with the most self-determined motivation on the left side of the SDT-continuum and the least on the right side. These subscales are: EM-external regulation, EM-introjected regulation, EM-identified regulation, and IM-intrinsic regulation. Scores are calculated as the average score on each subscale. Research has shown one of the drivers of PIF to be the internalization of motivation [7]. Thus, if the PIQ is a valid measure of professional identity, its total score should reflect some measure of this internalization; it should correlate positively with SRQ-A subscales representing internal qualities of motivation and, conversely, It should correlate negatively with SRQ-A subscales representing external qualities of motivation. For this analysis all three datasets (2015, 2016, and 2017) were merged in SPSS. Significance was tested in a two-tailed fashion. Normal distribution was checked using measures of skewness and kurtosis.

#### Internal consistency

Internal consistency of the questionnaire was tested using Cronbach’s alpha, where values of 0.7 and above were considered to reflect sufficient reliability.

## Results

In this section we first provide a description of the datasets for each respective analysis, for which descriptive statistics of the SRQ-A questionnaire are shown separately. What follows is an overview of the two factor analyses, the results of the Pearson’s correlations, the I-CVI, and lastly the results of the reliability analysis.

### Descriptives

Characteristics of the research population as well as the distribution of the data are described in Tables 1 and 2*.* Skewness and kurtosis measures were suggestive of non-normal distribution. The same held true for the distribution of average SQR-A scores for each of its subscales. Z-scores for skewness and excess kurtosis necessitated rejection of the normal distribution null-hypothesis in all four databases. Dataset 2015, dataset 2016, and the merged dataset 2015 + 2016 + 2017 were nevertheless of sufficient size to approximate normal distribution in accordance with the central limit theorem. Therefore cut-off points of ±2 for skewness and ± 7 for kurtosis were used [28]. Dataset 2017 did not meet these criteria and was therefore disregarded for factor analysis.

**Table 1:** Research population characteristics

	Dataset 2015	Dataset 2016	Dataset 2017	Merged dataset 2015 + 2016 + 2017
Response rate	38.96%	16.46%	13.08%	22.83%
Respondents PIQ (missing)	782 *(171)*	362 *(46)*	284 *(33)*	1420 *(247)*
Mean age (min-max)	22.09 *(17–39)*	21.78 *(17–33)*	22.10 *(17–32)*	22.01 *(17–39)*
Gender m/f (missing)	189/593 (52)	73/288 (16)	53/231 (14)	314/1105 (82)
PIQ mean/median (*std. deviation*)	38.77/39 *(5.12)*	38.95/40 *(5.40)*	38.36/39 *(5.40)*	38.73/39 *(5.25)*
PIQ Skewness (*std. error*)	−0.51 *(0.09)*	− 0.57 *(0.13)*	−0.75 *(0.15)*	− 0.58 *(0.07)*
PIQ Kurtosis (*std. error*)	0.90 *(0.18)*	0.47 *(0.26)*	1.19 *(0.29)*	0.85 *(0.13)*

*PIQ* Professional Identity Questionnaire

**Table 2:** Data distribution Academic Self-Regulation Scale (SRQ-A)

	Merged dataset 2015 + 2016 + 2017			
	External Regulation	Introjected Regulation	Identified Regulation	Intrinsic Motivation
SRQ-A mean/median (*std. deviation*)	1.61/1.50 *(0.68)*	2.21/2.25 *(0.85)*	4.26/4.25 *(0.53)*	4.32/4.25 *(0.56)*
SRQ-A Skewness (*std. error*)	1.24 *(0.06)*	0.40 *(0.06)*	−0.56 *(0.06)*	−1.02 *(0.06)*
SRQ-A Kurtosis (*std. error*)	1.31 *(0.13)*	−0.55 *(0.13)*	0.51 *(0.13)*	2.33 *(0.13)*

### Factor analyses

#### Exploratory factor analysis, confirmatory factor analysis & invariance analysis

The rotated factor matrices for both the EFA in dataset 2015 and the CFA in dataset 2016 are shown in Table 4.

The EFA uncovered two latent factors represented by the items of the PIQ. Items A through E loaded onto the first factor and items F through J loaded onto the second factor. This followed positive and negative wording of the first and second set of items, respectively.

In order to construct a model that best fits the data, three CFA models were built. Model I featured two factors, in concurrence with EFA findings, it also allowed for correlation between these factors and items were specifically designated to each factor in accordance how they loaded in the EFA. Model II was identical to model I except that it allowed for the covariance of errors between items I and G. Model III, in its turn, was identical to model II except that item J was removed from it. Table 3 shows each model with its corresponding model fit indices. Lastly, multigroup Confirmatory Factor Analysis revealed full configural and metric equivalence while model fit was seen to increase for a model in which the intercept of item D was allowed to vary between genders (χ^2^ = 6.64, *p* < .01), indicating partial scalar equivalence.

**Table 3:** CFA models & their fit indices

	Model I	Model II	Model III
CFI^*a*^	0.945	0.976	0.981
TLI^*b*^	0.929	0.967	0.973
RMSEA^*c*^	0.080	0.055	0.053
SRMR^*d*^	0.113	0.043	0.040

^*a*^*threshold = 0.95*

^*b*^*threshold = 0.95*

^*c*^*threshold = 0.06*

^*d*^ *threshold = 0.05*

Hooper et al. [10]. Structural Equation Modeling: Guidelines for Determining Model Fit. Electronic Journal on Business Research Methods, 6(1), 53–60

The factor loadings shown in Table 4 correspond to those of model II. CFA loadings can be seen to run parallel to the EFA loadings, with the exception of items G and J. Both EFA and CFA showed poor factor loading of item J.

**Table 4:** Factor analysis & Inter item Content Validity Index (I-CVI

PIQ Item	Scoring	EFA Dataset 2015		CFA II Dataset 2016		I-CVI**
		Factor 1	Factor 2	Factor 1	Factor 2	
A: I am a person who considers the doctors’ group important.		0.630*	0.164*	0.650		0.50
B: I am a person who identifies with the doctors’ group.		0.825*	0.130*	0.829		1.00
C: I am a person who feels strong ties with the doctors’ group.		0.788*	0.086*	0.852		1.00
D: I am a person who is glad to belong to the doctors’ group.		0.718*	0.176*	0.799		1.00
E: I am a person who sees myself belonging to the doctors’ group.		0.614*	−0.012*	0.623		1.00
F: I am a person who makes excuses for belonging to the doctors’ group.	R	0.011*	0.754*		0.737	0.50
G: I am a person who tries to hide belonging to the doctors’ group.	R	0.074*	0.800*		0.634	0.50
H: I am a person who feels held back by the doctors’ group.	R	0.148*	0.715*		0.736	0.25
I: I am a person who is annoyed to say that I’m a member of the doctors’ group.	R	0.112*	0.808*		0.730	0.00
J: I am a person who criticizes the doctors’ group.	R	0.117	0.381		0.482	0.25

Notes: R indicates items with reverse scoring

* indicates statistical significance (*p* ≤ 0.05)

** I-CVI score computed as the number of experts rating 3–4 divided by the total number of experts

### Pearson’s correlations between variables

Table 5 shows the correlations between the total score of the PIQ and the average scores of each subscale of the SRQ-A. Except EM-introjected regulation, each subscale correlated significantly with the PIQ total score, with negative correlation on the less self-determined side of the spectrum and positive correlation on the more self-determined side.

**Table 5:** Pearson’s correlations

SRQ-A subscale	N	PIQ total score Pearson’s correlation	p*
External regulation	*1391*	*−0.13*	*0.00*
Introjected regulation	*1392*	*−0.04*	*0.18*
Identified regulation	*1387*	*0.35*	*0.00*
Intrinsic motivation	*1384*	*0.37*	*0.00*

* significance threshold = p ≤ 0.05

Note: SRQ-A - Academic Self-regulation Scale of motivation

### Inter item content validity index

Table 4 shows the results of the I-CVI filled in by the experts. All experts responded to the email, four of them filled in the I-CVI. One expert declined participation in the study because she did not believe professional identity could be measured quantitatively. Four out of ten items were unanimously deemed relevant by the experts (Items B, C, D, and E). Item I was the only item to receive a unanimous non-relevant scoring. Three out of four experts agreed with the statement:

“*Regarding the questionnaire as a whole, I find some facets of professional identity unrepresented by the items*.”

When asked to describe or name what they felt was missing from the PIQ, they provided the following answers:

E1: “*I miss aspects of the process leading to PI in the questions.*“

E2: “*I found some of the items didn't have much to do with identity - there are lots of various models of identity and each describes identity differently so without ascribing to one I cannot say what is missing but would recommend you do that.*”

E3: “*Although I am not officially registered as a doctor, I feel I belong to the doctors' group.*”

E4: “*A sense of belonging and emotional attachment to the doctor’s profession is well represented in the items. I do miss items representing a self-understanding as a doctor, which is a central feature of identity in the literature. Furthermore, I have some questions about the scaling; it now represents frequency (never --- very often). Why was frequency chosen, and not the extent to which students identify with the doctor’s profession (not at all --- very much)?*”

### Cronbach’s alpha

The internal consistency analysis showed a Cronbach’s alpha value of 0.82 for reliability, which is well above the cut-off value of 0.70, implying good consistency between the items of the PIQ.

## Discussion

This study presents support for the validity and reliability of using Brown et al.’s [5] Professional Identity Questionnaire as a tool for the measurement of professional identity among medical students, using the APA, AERA and NCME’s account of validity [12]. That is, findings from our factor analyses, including measurement invariance, Pearson’s correlations tests and Cronbach’s alpha as a reflection of its internal consistency provide indications for its valid and reliable use in this context. However, evaluation of the instrument by experts on professional identity formation reveals some shortcomings which should be appeased.

EFA and CFA uncovered two latent factors underlying the items of the PIQ. Rather than reflecting specific attributes of medical doctors like the five factors underlying Tagawa’s recently developed scale [2019], these two factors seem to reflect attached and detached attitudes towards the medical profession, as evidenced by the mirrored factor loading on positively worded items A through E and negative worded items F through J, respectively. We propose to label these factors as: PIF-attachment and PIF-detachment. Building on the CFA, invariance analysis revealed how this model of the PIQ upholds configural and metric equivalence between genders. These findings reflect the psychometric equivalence of the PIQ’s measure of PIF across genders and, as such, educators may freely administer the measure to male and female students without having to account for differences in interpretation. To this must be added that, due to its partial scalar equivalence, researchers using the PIQ as a measurement model in the context of a larger structural equation modelling analysis are advised to allow the intercept of item D to vary across gender.

Pearson’s correlation of the PIQ with the SRQ-A –a validated tool for the measurement of quality of motivation– revealed a spectrum of correlations. In concurrence with Ryan & Deci’s Self-Determination theory [2000], the PIQ’s total score showed a progressively positive correlation with the increasingly intrinsic qualities of motivation represented by the SRQ-A’s subscales. The absence of a statistically significant correlation with introjected regulation can either be an indication of the PIQ failing to include acting out of a sense of obligation in its negative measure of PIF, or it could mean that this quality of motivation has little relevance among medical students in this respect.

Four experts rated four of the ten items on the PIQ as relevant using an Inter item Content Validity Index. They stated missing non-affective aspects of identity, like ‘self-understanding’, and items representing developmental stages of PIF, as well as items professing an explicit sense of belonging to the doctor’s group in spite of a lack of formal inclusion. One expert, furthermore, posited that the rating scale of the PIQ should be altered to reflect intensity rather than frequency. Modification of the PIQ in order to account for these omissions seems warranted.

Kalet et. al. [11] have described the process of professional identity formation through a qualitative lens, as one featuring frequent lapses and bumps along the road. However, their method is time-intensive and therefore difficult to scale-up. Due to its quantitative character, the PIQ can be more easily applied to a large scale, allowing for the survey of a large body of students that is less time-intensive. In addition, the PIQ’s measure of PIF-attachment as well as detachment further widens its utility; it allows educators to gauge not only the beneficial but also the undesirable effects their curriculum has on students’ PIF.

### Study limitations & recommendations for use of the PIQ in further studies

In advance of its implementation into medical education, two alterations to the PIQ can be made in order to improve its validity as an instrument measuring PIF (Additional file 1: Appendix C), in addition to a second validity study in order to verify their effectiveness. The first entails removing item J in lieu of its weak factor loading and replacing it with an item reflecting belonging to the doctor’s profession out of a sense of obligation. This ensures the inclusion of an introjected quality of motivation. The second entails altering the rating scale of the PIQ from one representing frequency to one representing intensity, as per expert recommendation. In addition, the two factors can be labelled as PIF-attachment and PIF-detachment.

The decision to focus our investigation on content, internal structure and concurrent validity has left other dimensions of construct validity unexamined, these include response process validity, predictive validity, and consequential validity. These dimensions anchor the instrument in the practical world, emphasizing the thoughts going through students’ heads when it is administered, any consequences its administration might have, and its predictive value in terms of a defined criterion. Their omission here may have resulted in too theoretical an argument for the PIQ’s validity. As regards the I-CVI, the small pool of four experts meant that, in order to pass the test of content validity, each item had to be scored positively in a unanimous way. This effectively dichotomized each item into a pass or fail rather than resulting in an index, thereby removing nuance from the evaluation.

In contrast to these blind spots, the number and variety of students included in the final sample size adds weight to the findings presented in this article. Even so, the response rate was very low, opening the study up to nonresponse bias. Such bias can influence the interpretation of the study’s findings in the case of an unequal distribution of nonresponse in relation to the outcome parameter. That is, if professional identity itself may have influenced students’ decision to participate in the survey, the findings presented here may relate not to medical students as a whole, but only to those whose level of professional identity prompted them to participate in the study.

In concurrence with these limitations, future research might improve on this study by charting students’ reason for nonresponse, by including doctors and medical educators in the sample, by enlisting the aid of a larger pool of experts, and by including evidence on response process validity, predictive validity, and consequential validity.

## Conclusion

The PIQ can be effectively used as a quantitative measure of professional identity formation among medical students In this medical educational setting, it could provide educators with the feedback they need to improve their curriculum without the requirement of time-intensive qualitative approaches. Our findings indicate the PIQ’s measure of professional identity to be more nuanced than we initially hypothesized, including detached as well as attached attitudes towards the medical profession. We argue that this adds rather than subtracts to the argument for its validity but future researchers might nevertheless add to this body of evidence for the validity and reliability of the PIQ in a number of ways, as described in the limitations section of this study. As it stands, however, the PIQ, posing as one among a number of approaches, provides another piece of the puzzle through which medical educators can harness the powers of quantitative analysis in their quest to craft the doctors of tomorrow.

## Supplementary Information

**Additional file 1.**

## Data Availability

The datasets during and/or analysed during the current study available from the corresponding author on reasonable request.
