# Correction to: Validation of the professional identity questionnaire among medical students

**DOI:** 10.1186/s12909-022-03160-w

**Published:** 2022-02-16

**Authors:** Daan Toben, Marianne Mak-van der Vossen, Anouk Wouters, Rashmi A. Kusurkar

**Affiliations:** 1 Faculty of Health and Life Sciences, Vrije Universiteit Amsterdam, Polanenstraat 54F, 1013, VZ, Amsterdam, The Netherlands; 2 Amsterdam UMC, Faculty of Medicine Vrije Universiteit Amsterdam, Research in Education, Amsterdam, the Netherlands; 3 LEARN! Research Institute for Learning and Education, Faculty of Psychology and Education, VU University Amsterdam, Amsterdam, the Netherlands

**Correction to: BMC Med Educ 21, 359 (2021)**

**https://doi.org/10.1186/s12909-021-02704-w**

Following publication of the original article [1], we have been informed that first name and last name have been swapped for 1st, 2nd and 3rd author.

The original article has been corrected.
