# Supplementary material for: Validation of the professional identity questionnaire among medical students
Source: BMC Med Educ. 2021 Jun 28;21:359. doi: 10.1186/s12909-021-02704-w (PMC8240200; doi:10.1186/s12909-021-02704-w)
Supplement: Supplementary file 1 — Additional file 1. [file 12909_2021_2704_MOESM1_ESM.docx]

**Appendix A**

The Professional Identity Questionnaire

**Professional Identity Questionnaire**

**Please indicate on scale of 1 to 5 how much the given statements are true for you.**

**Never                Seldom Sometimes              Often Very Often**

**1                         2       3 4                     5**

1. I am a person who considers the doctors’ group important. 1 – 2 – 3 – 4 - 5
2. I am a person who identifies with the doctors’ group. 1 – 2 – 3 – 4 - 5
3. I am a person who feels strong ties with the doctors’ group. 1 – 2 – 3 – 4 - 5
4. I am a person who is glad to belong to the doctors’ group. 1 – 2 – 3 – 4 - 5
5. I am a person who sees myself belonging to the doctors’ group. 1 – 2 – 3 – 4 - 5
6. I am a person who makes excuses for belonging to the doctors’ group. 1 – 2 – 3 – 4 - 5
7. I am a person who tries to hide belonging to the doctors’ group. 1 – 2 – 3 – 4 - 5
8. I am a person who feels held back by the doctors’ group. 1 – 2 – 3 – 4 - 5
9. I am a person who is annoyed to say that I’m a member of

the doctors’ group. 1 – 2 – 3 – 4 - 5

1. I am a person who criticizes the doctors’ group. 1 – 2 – 3 – 4 – 5

**Appendix B-I**

The SRQ-A (Dutch version)

| SRQ-A – Redenen om te studeren voor de geneeskundeopleiding |
| --- |

Studenten kunnen verschillende redenen hebben om te studeren. Geef aan in welke mate de volgende redenen voor jou belangrijk zijn om te studeren.

(Regulatietype + nummer; External Regulation = ER 1 t/m 4, Introjected Regulation = InR 1 t/m 4, Identified Regulation = IdR 1 t/m 4 en Intrinsic Motivation = IM 1 t/m 4)

□ Helemaal niet □ Niet zo □ Neutraal □ Belangrijk □ Heel belangrijk
belangrijk belangrijk

1 2 3 4 5

Ik ben gemotiveerd om te studeren voor geneeskunde omdat…

1) IdR1 … ik nieuwe dingen wil bijleren.

2) InR2 … ik me schuldig zou voelen als ik het niet zou doen.

3) ER1 … ik verondersteld word dit te doen.

4) IM2 … geneeskunde studeren leuk is.

5) IdR4 … ik dit een belangrijk levensdoel vind.

6) IM4 … ik geneeskunde studeren een aangename bezigheid vind.

7) InR3 … ik me zou schamen als ik het niet zou doen.

8) ER3 … anderen (ouders, vrienden, docenten,…) me hiertoe verplichten.

9) InR4 … ik anderen de indruk wil geven dat ik een goede student ben.

10) IM3 … ik geneeskunde studeren boeiend vind.

11) IdR2 … ik geneeskunde persoonlijk zeer waardevol vind.

12) IdR3 … dit voor mij een persoonlijk belangrijke keuze is.

13) ER4 … anderen (ouders, vrienden, docenten,…) dit van mij verwachten.

14) IM1 … geneeskunde studeren me erg interesseert.

15) ER2 … anderen (ouders, vrienden, docenten,…) me dwingen om dit te doen.

16) InR1 … ik wil dat anderen denken dat ik verstandig ben.

**Appendix B-II**

The SRQ-A (English version)

| SRQ-A – Reasons for studying medicine |
| --- |

The follow questionnaire measures your motivation for studying medicine. Please indicate how

important each of the listed motives is for you to study

(Regulatietype + items; External Regulation = ER 1 t/m 4, Introjected Regulation = InR 1 t/m 4, Identified Regulation = IdR 1 t/m 4 en Intrinsic Motivation = IM 1 t/m 4)

□ Completely not □ Rather not □ Neutral □ Important □ Very important
important important

1 2 3 4 5

Why are you studying medicine? I am studying…

1) IdR1 … because I want to learn new things.

2) InR2 … because I would feel guilty if I wouldn’t do so.

3) ER1 … because I’m supposed to do so.

4) IM2 … because I enjoy studying medicine.

5) IdR4 … because this is an important life goal to me.

6) IM4 … because studying medicine is an exciting thing to do.

7) InR3 … because I would feel ashamed if I wouldn’t do so.

8) ER3 … because others (parents, friends, etc.) oblige me to do so.

9) InR4 … because I want others think I’m a good student.

10) IM3 … because studying medicine is fun.

11) IdR2 … because studying medicine is personally important to me.

12) IdR3 … because this represents a meaningful choice to me.

13) ER4 … because that’s what others (parents, friends, etc.) expect me to do.

14) IM1 … because I am highly interested in studying medicine.

15) ER2 … because that’s something others (parents, friends, etc.) force me to do.

16) InR1 … because I want others to think I’m smart.

**Appendix C**

The Professional Identity Questionnaire revised

**Professional Identity Questionnaire**

**Please indicate on scale of 1 to 5 to what extent you agree with the given statements.**

**Strongly disagree Disagree Neutral Agree Strongly agree**

**1 2 3 4                     5**

1. I am a person who considers the doctors’ group important. 1 – 2 – 3 – 4 - 5
2. I am a person who identifies with the doctors’ group. 1 – 2 – 3 – 4 - 5
3. I am a person who feels strong ties with the doctors’ group. 1 – 2 – 3 – 4 - 5
4. I am a person who is glad to belong to the doctors’ group. 1 – 2 – 3 – 4 - 5
5. I am a person who sees myself belonging to the doctors’ group. 1 – 2 – 3 – 4 - 5
6. I am a person who makes excuses for belonging to the doctors’ group. 1 – 2 – 3 – 4 - 5
7. I am a person who tries to hide belonging to the doctors’ group. 1 – 2 – 3 – 4 - 5
8. I am a person who feels held back by the doctors’ group. 1 – 2 – 3 – 4 - 5
9. I am a person who is annoyed to say that I’m a member of

the doctors’ group. 1 – 2 – 3 – 4 - 5

1. I am a person who would feel guilty for not belonging to the

doctor’s group 1 – 2 – 3 – 4 - 5
